# Supplementary material for: MDF Regulates a Network of Auxin‐Dependent and Auxin‐Independent Pathways of Adventitious Root Regeneration in Arabidopsis
Source: Plant Direct. 2025 Apr 23;9(4):e70050. doi: 10.1002/pld3.70050 (PMC12018534; doi:10.1002/pld3.70050)

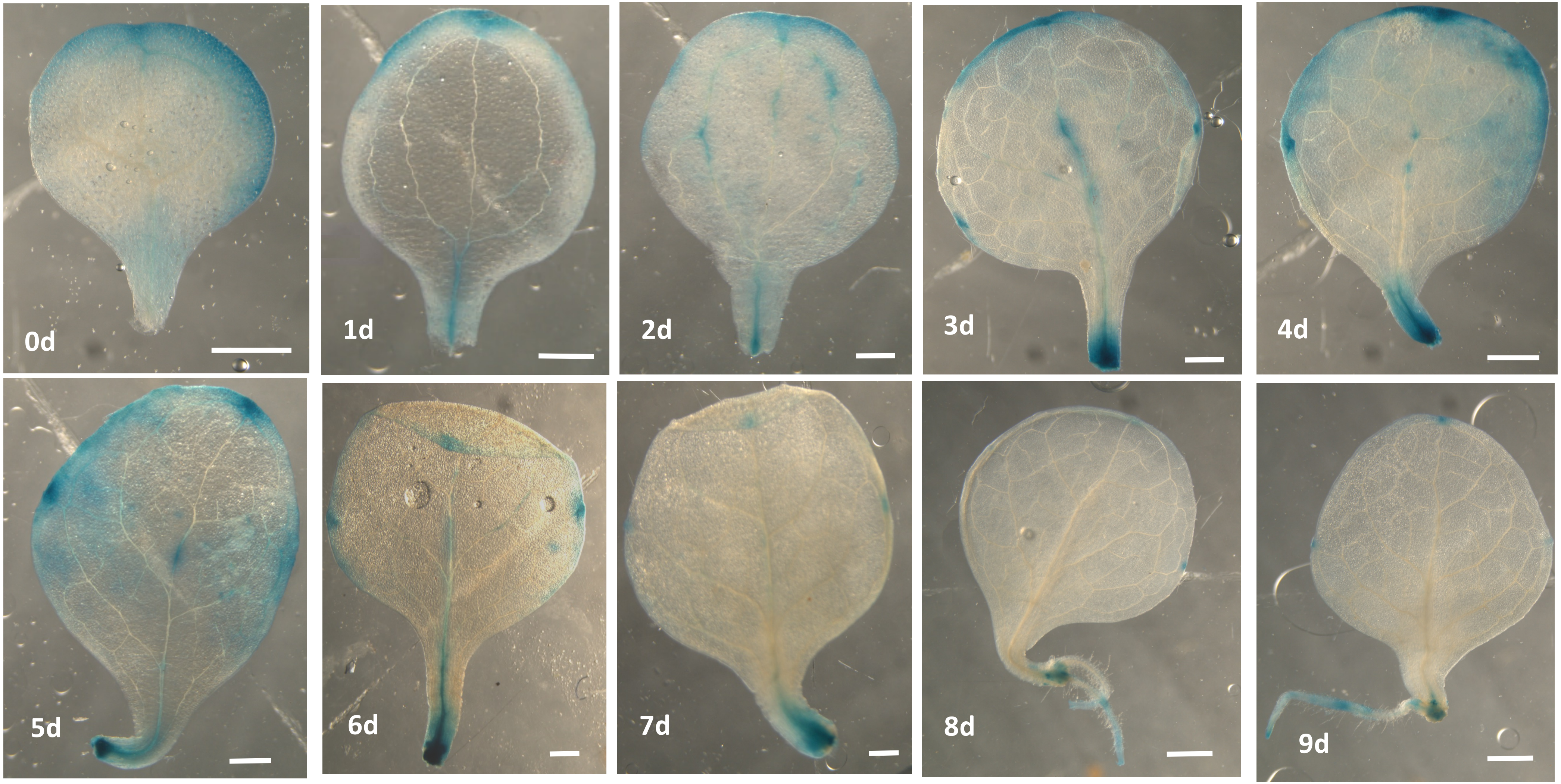

Fig. S1. Expression of the auxin reporter *DR5::GUS* during *Arabidopsis* adventitious root regeneration. Scale bars =500 um.

Fig. S2. *PIN1* expression in *Arabidopsis* leaf and regenerating root.

A

B

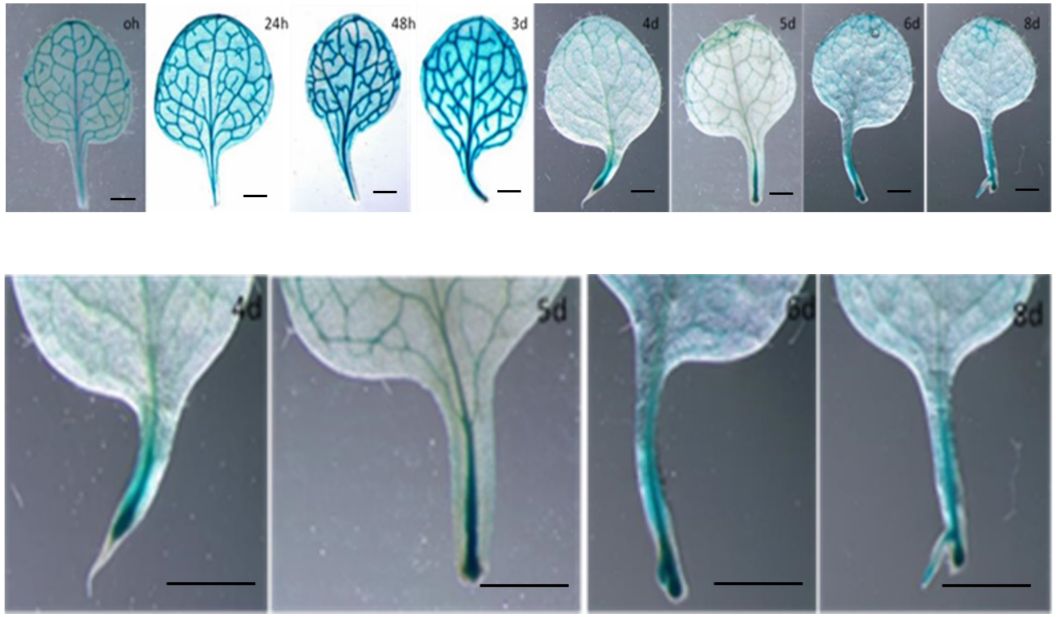

(A) *PIN1::GUS* expression in leaf of Arabidopsis when cultured from 0 h to 8 d, with scale bars = 1 mm. (B) Magnification of images in A, showing PIN1::GUS expression in petiole of leaf of Arabidopsis from 4 d to 8 d, with scale bars = 0.5 mm.

C

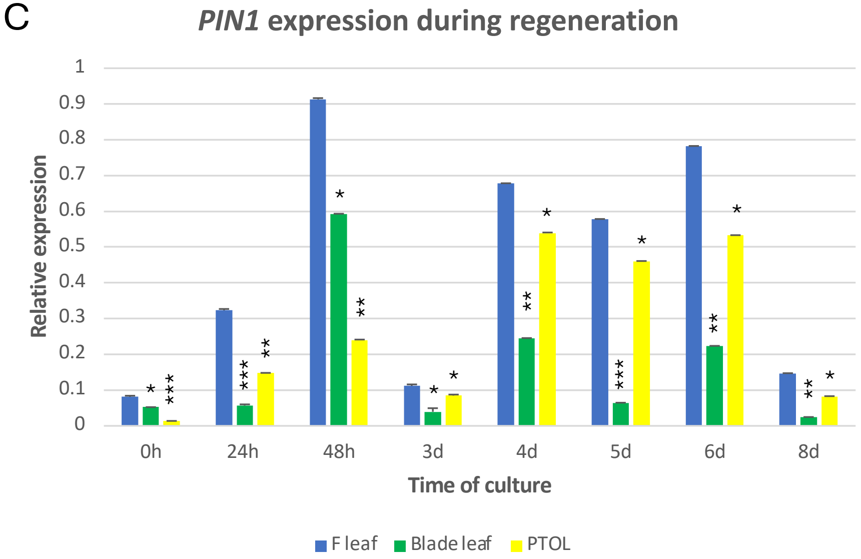

C. Relative expression of the *PIN1* gene analysed using qRT-PCR in whole leaf (blue bars, F leaf), leaf blade (green bars) and petiole (yellow bars, PTOL) from 0 h to 8 d by using the *UBC* reference gene. Values represents means and error bars are SEM (n = three biological repeats with three technical repeats). Statistical significance was determined using Student's t-test for independent samples compared to wild type values.

Fig. S3. *PIN3* expression in *Arabidopsis* leaf and regenerating root.

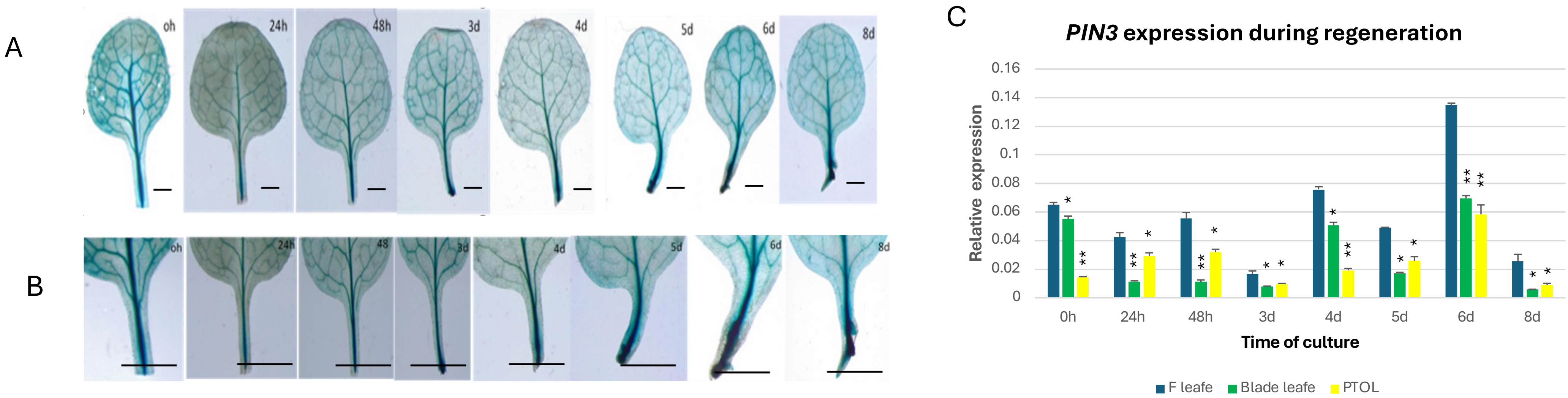

(A) *PIN3::GUS* expression in leaf of Arabidopsis when cultured from 0 h to 8 d, with scale bars = 1mm. (B) Magnification of images in A, showing *PIN3::GUS* expression in petiole of leaf of Arabidopsis from 0 h to 8 d, with scale bars = 0.5 mm.

Relative expression of the *PIN3* gene analysed using qRT-PCR in whole leaf (blue bars), leaf blade (green bars) and petiole (yellow bars, PTOL) from 0 h to 8 d using the *UBC* reference gene. Values represent means and error bars are SEM (n = three biological repeats with three technical repeats). Statistical significance was determined using Student's t-test for independent samples compared to wild type values.

S4. *PIN7::GUS* expression in *Arabidopsis* leaf and regenerating root.

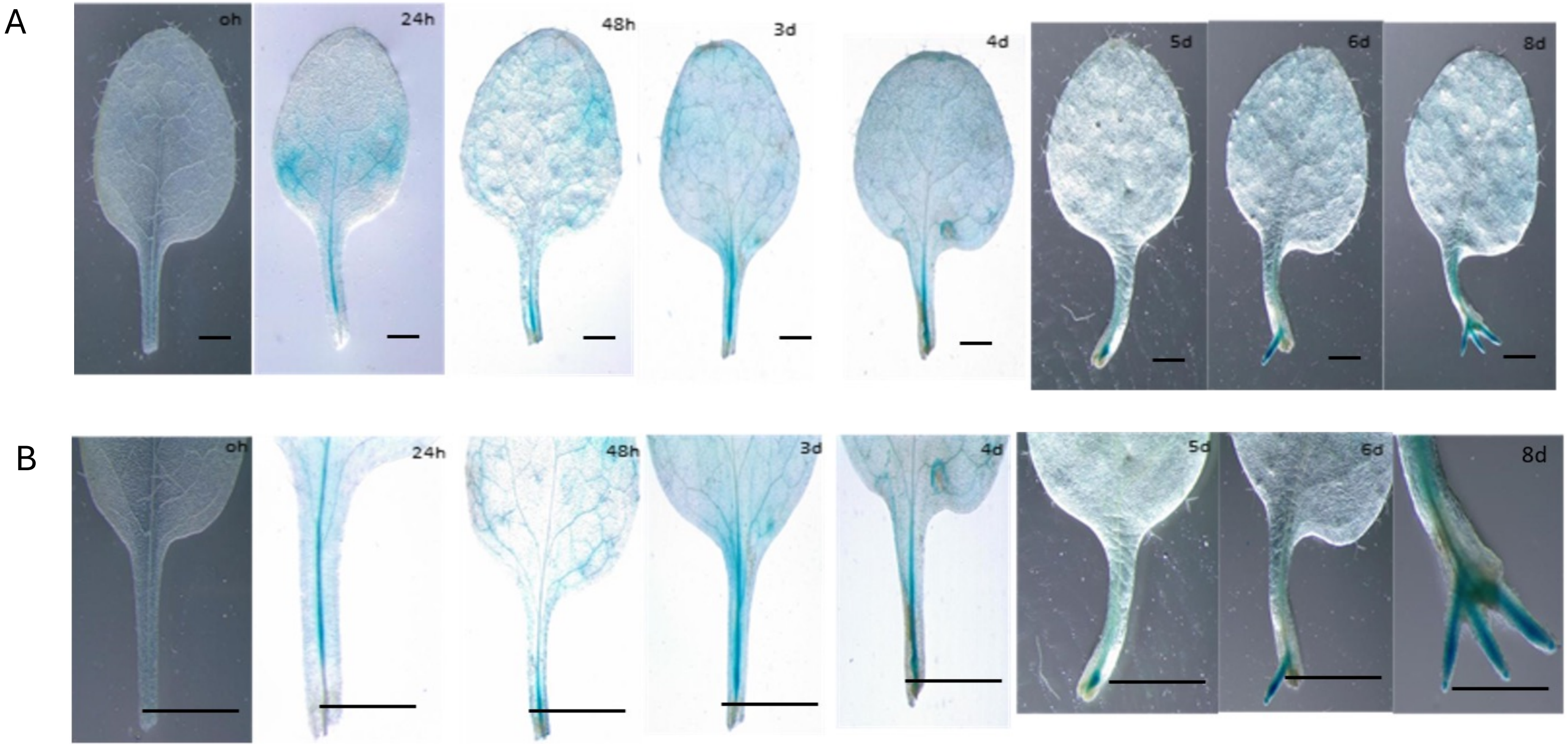

(A) *PIN7::GUS* expression in leaf of Arabidopsis when cultured from 0 h to 8 d, with scale bars = 1 mm. (B) Magnification of images in A, showing *PIN7::GUS* expression in petiole of leaf of Arabidopsis from 0 h to 8 d, with scale bars = 0.5 mm.

Fig. S5. *VAMP714::GUS* expression in *Arabidopsis* leaf and regenerating root.

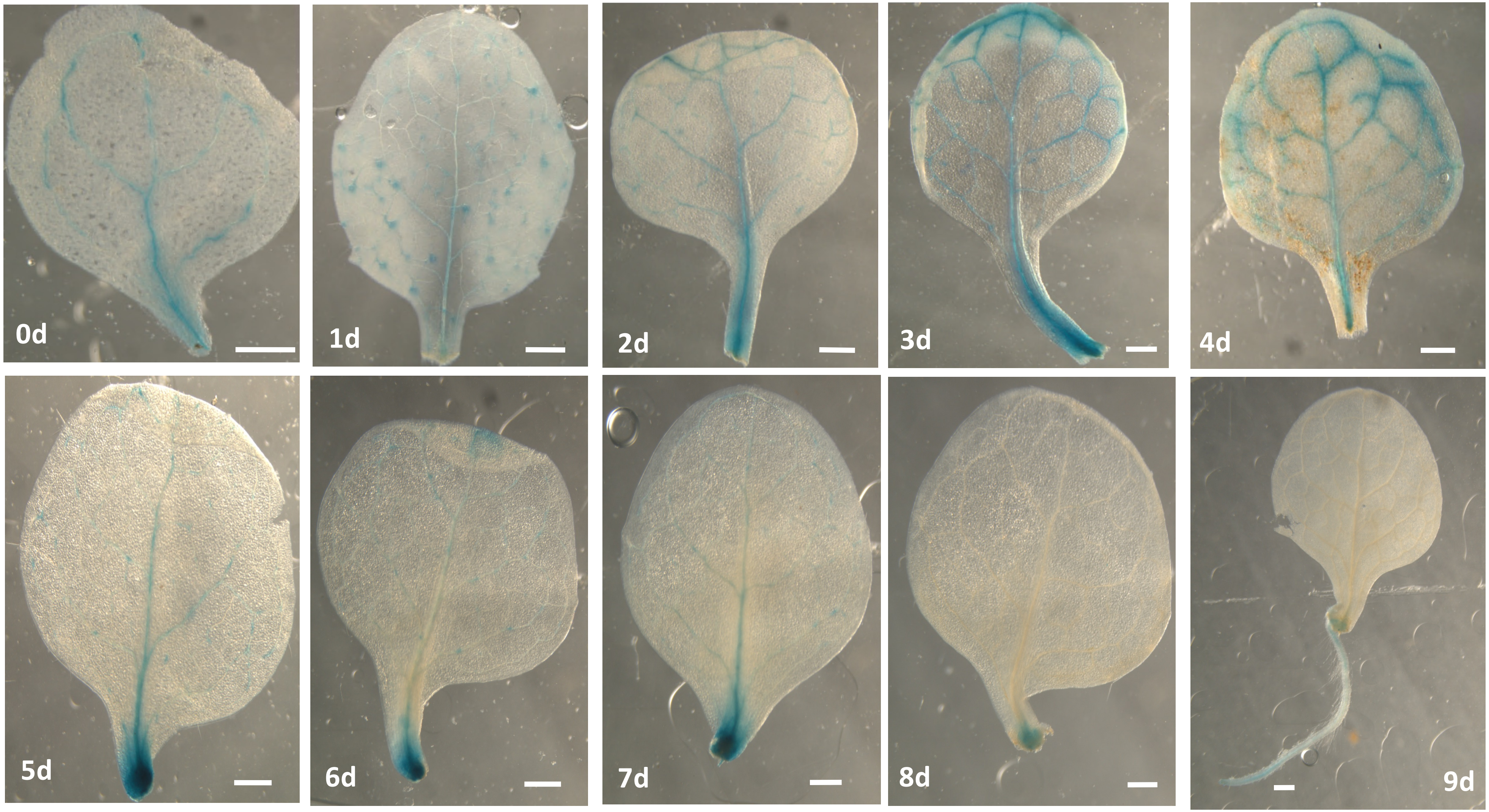

scale bars = 500 um

Fig. S6. *EBS::GUS* expression in *Arabidopsis* leaf and regenerating root.

A

B

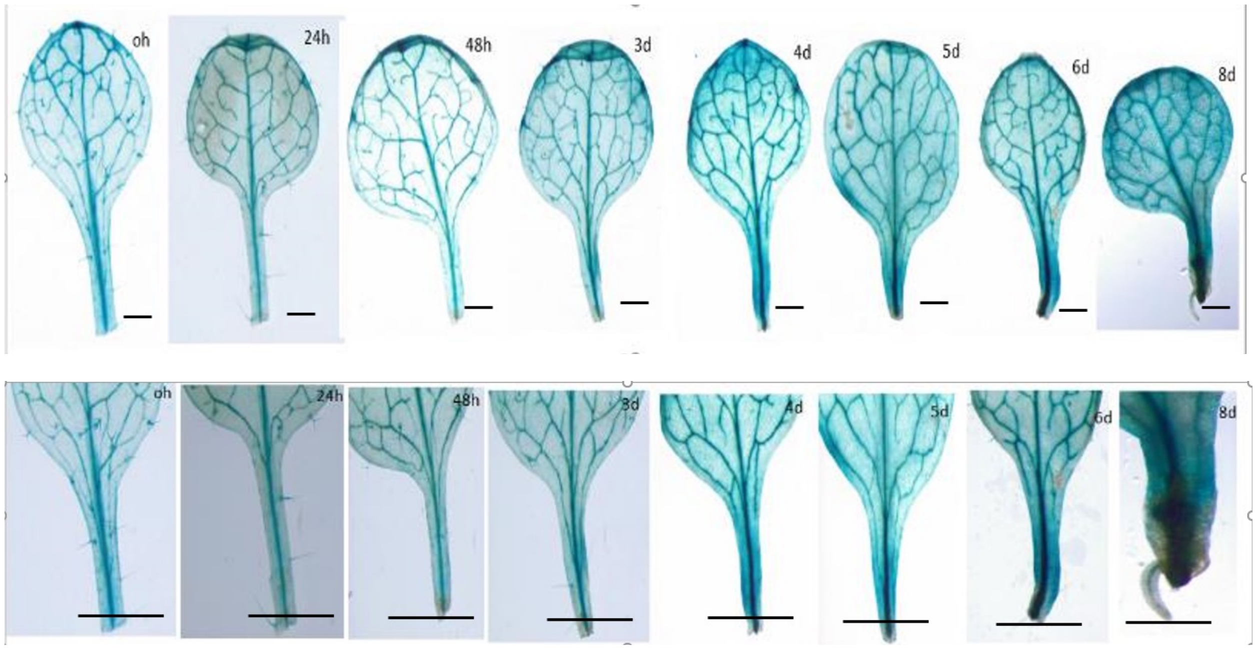

Fig. S6. A) *EBS::GUS* expression in leaf of Arabidopsis when cultured from 0 h to 8 d, with scale bars = 1 mm. (B) Magnification of images in A, showing *EBS::GUS* expression in petiole of leaf of Arabidopsis from 0 h to 8 d. Scale bars = 0.5 mm.

MDF (AT5G16780) expression in *Arabidopsis thaliana* detached leaves cultured on sucrose-free B5 medium (mock, without NPA) at 22°C

A

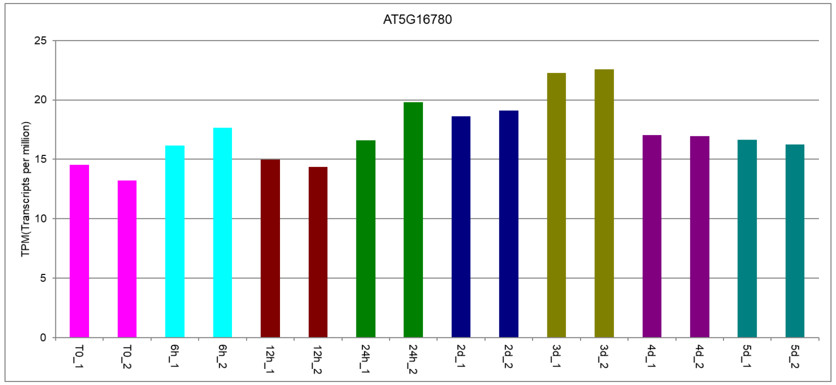

MDF (AT5G16780) expression in *Arabidopsis thaliana* detached leaves cultured on sucrose-free B5 medium with NPA at 22°C

B

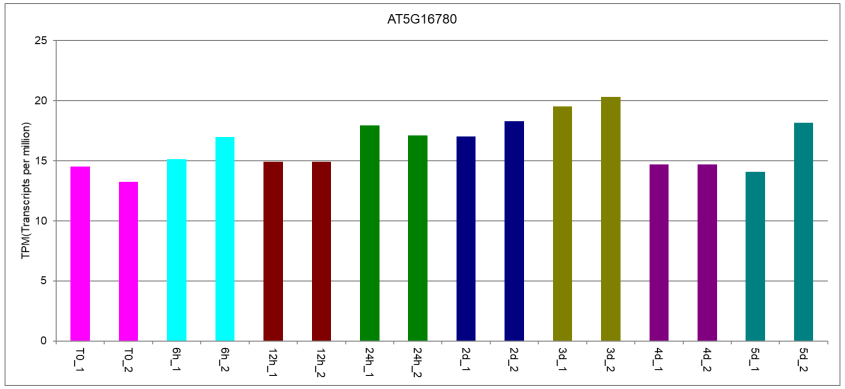

RAP2.7 (AT2G28550) expression in *Arabidopsis thaliana* detached leaves cultured on sucrose-free B5 medium (mock, without NPA) at 22°C

C

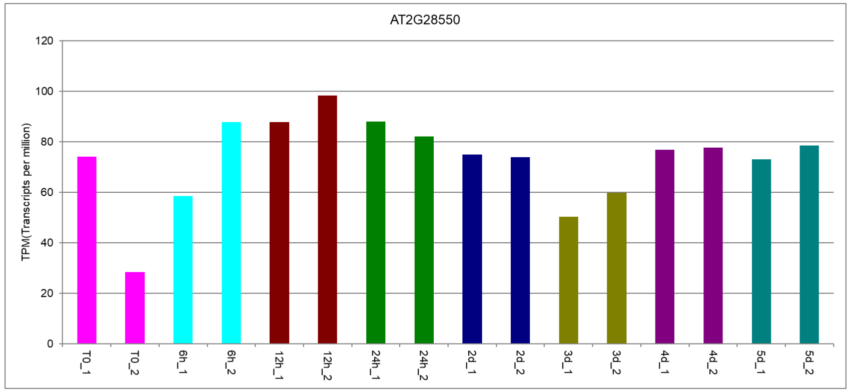

RAP2.7 (AT2G28550) expression in *Arabidopsis thaliana* detached leaves cultured on sucrose-free B5 medium with NPA at 22°C

D

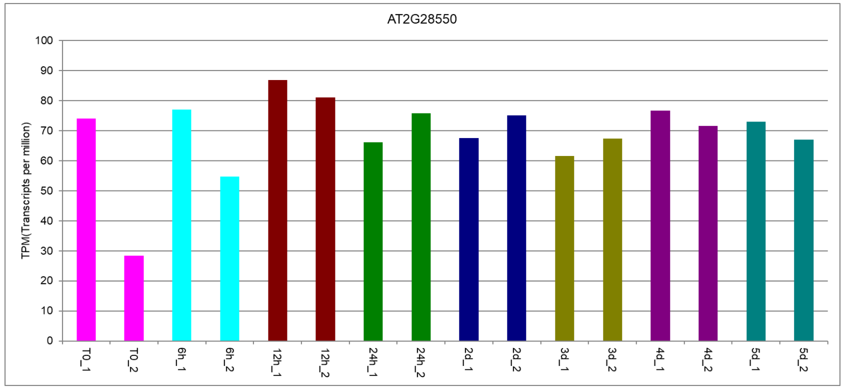

Fig. S7

Fig. S7. Expression of *MDF* and *RAP2.7* genes as determined by RNA-seq over a 5 day leaf culture period for adventitious root regeneration. Bars of the same colour represent biological replicates, at 0 h (T0, pink bars), 6 h (pale blue bars) 12 h (brown bars), 24 h (green bars), 2 d (dark blue bars), 3 d (olive green bars), 4 d (purple bars) and 5 d (teal bars).
Data from Liu et al. (2022) Plant Comms. 3,100306.

Fig. S8. *MDF* inducible expression

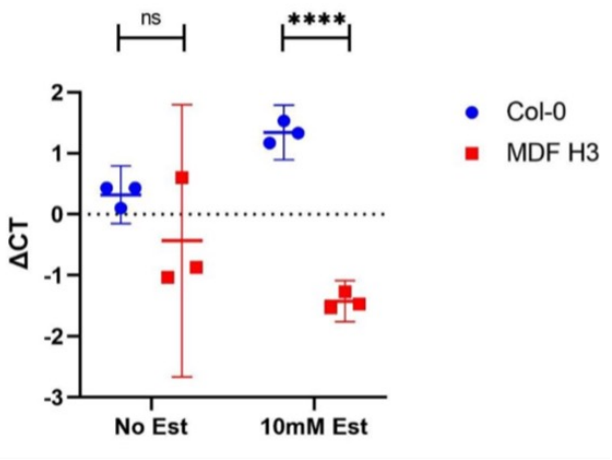

qRT-PCR analysis of *MDF* gene expression in wild type and MDF-OV. Using UBC reference gene was quantified without treatment and after treatment with 10 mM estradiol. t-test with no treatment ($p = 0.28$, df: 2.180); 10 mM estradiol (****p= 0.000051, df 3.720). Data from Thompson et al. (2023).

Delta-delta Ct calculation: No estradiol induction

**Sample**	**Gene of interest (*MDF*)**	**Housekeeping gene (*UBC*)**			**Relative fold change**
	Average Ct	Average CT	ΔCt	ΔΔCt	2^-(ΔΔCt)
**Col-0 A**	20.73	20.30	0.43	0.11	0.92
**Col-0 B**	21.60	21.17	0.43	0.11	0.92
**Col-0 C**	21.47	21.37	0.10	-0.22	1.16
**MDF-H3 A**	22.10	21.50	0.6	0.28	0.82
**MDF-H3 B**	20.53	21.40	-0.8667	-1.19	2.28
**MDF-H3 C**	20.93	21.97	-1.0333	-1.35	2.56

Delta-delta Ct calculation: Induction with 10 mM estradiol

**Sample**	**Gene of interest (*MDF*)**	**Housekeeping gene (*UBC*)**			**Relative fold change**
	Average Ct	Average CT	ΔCt	ΔΔCt	2^-(ΔΔCt)
**Col-0 A**	24.2	22.67	1.53	0.19	0.87
**Col-0 B**	23.93	22.6	1.333	-0.01	1
**Col-0 C**	23.53	22.37	1.1667	-0.17	1.13
**MDF-H3 A**	21.43	22.7	-1.2667	-2.61	6.09
**MDF-H3 B**	21.27	22.73	-1.4667	-2.81	7
**MDF-H3 C**	21.1	22.63	-1.533	-2.87	7.33

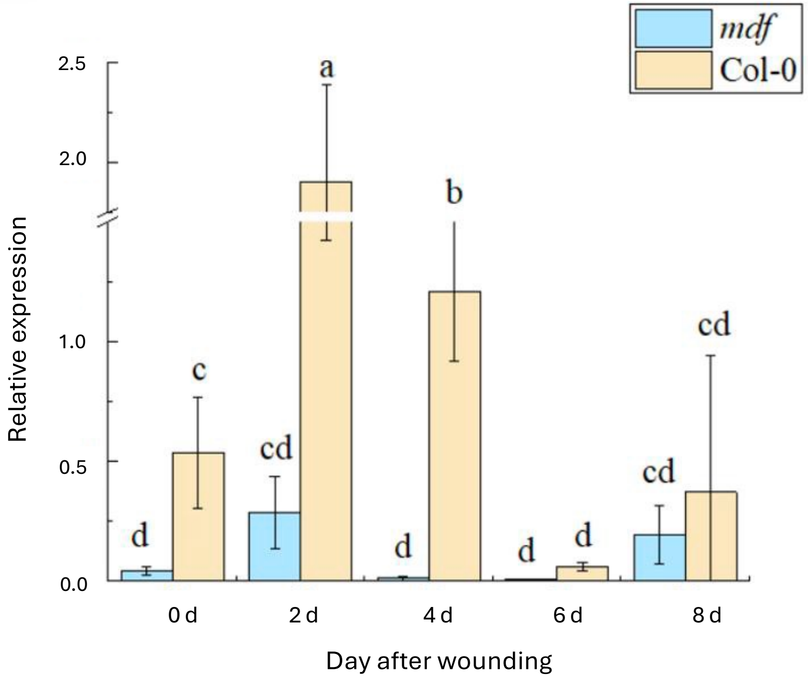

Fig. S9. Relative expression of *IAA1* in *mdf* mutant and WT (Col-0) analysed using qRT-PCR in whole leaf at 0d, 2d, 4d, 6d, 8d using *ACTIN2* as the reference gene. Values represent means and error bars are SEM (n = three biological repeats with three technical repeats). One-way ANOVA and correlation analysis was performed using SPSS 26.0 software, and Duncan 's new multiple range method was used for significance test ($p< 0.05$).

Fig. S10. *RAP2.7* expression levels in *35S::RAP2.7* transgenics. Bars represent SE of mean, n = 3.

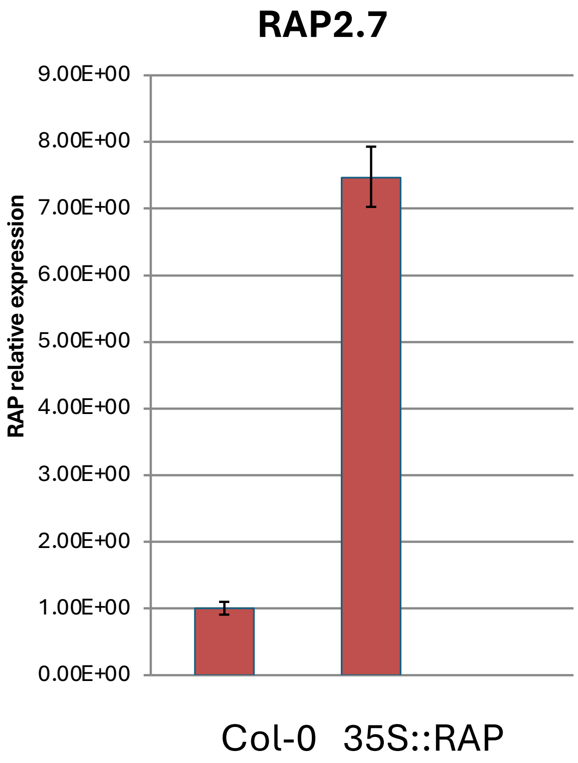

Fig. S11. *NAC1* expression levels in *35S:NAC1* transgenics. Bars represent SE of mean, n = 3.

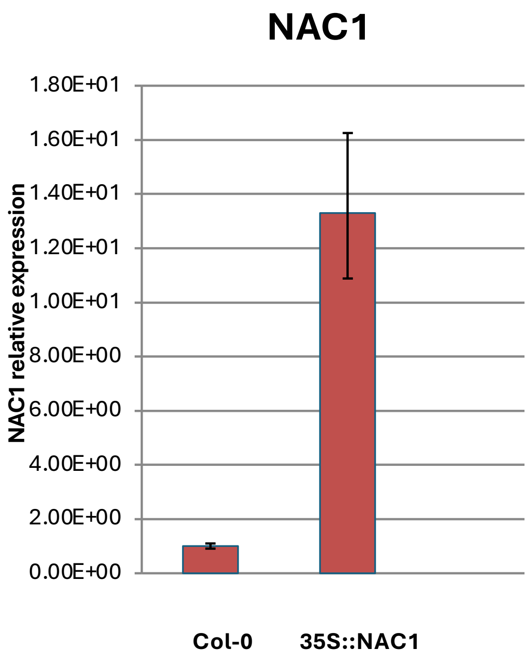

Supplement: Supplementary file 1 — Figure S1 Expression of the auxin reporter DR5::GUS during Arabidopsis adventitious root regeneration. Figure S2. PIN1 gene expression in Arabidopsis leaf and regenerating root. Figure S3. PIN3 gene expression in Arabidopsis leaf and regenerating root. Figure S4. PIN7 gene expression in Arabidopsis leaf and regenerating root. Figure S5. VAMP714::GUS gene expression in Arabidopsis leaf and regenerating root. Figure S6. EBS::GUS gene expression in Arabidopsis leaf and regenerating root. Figure S7. Expression of MDF and RAP2.7 genes as determined by RNA‐seq over a 5 day leaf culture period for adventitious root regeneration. Figure S8. MDF inducible gene expression. Figure S9. Relative expression of IAA1 in mdf mutant and WT (Col‐0) analyzed using qRT‐PCR in whole leaf at 0d, 2d, 4d, 6d, 8d using ACTIN2 as the reference gene. Figure S10. RAP2.7 expression levels in 35S::RAP2.7 transgenics. Figure S11. NAC1 expression levels in 35S:NAC1 transgenics. [file PLD3-9-e70050-s003.pdf]
