# Supplementary material for: MDF Regulates a Network of Auxin‐Dependent and Auxin‐Independent Pathways of Adventitious Root Regeneration in Arabidopsis
Source: Plant Direct. 2025 Apr 23;9(4):e70050. doi: 10.1002/pld3.70050 (PMC12018534; doi:10.1002/pld3.70050)
Supplement: Supplementary file 2 — Table S1 Gene expression data for the MDF gene regulation in response to hormones. [file PLD3-9-e70050-s004.docx]

**Suppl. Table S1.**

Gene expression data for the *MDF* gene regulation in response to

hormones obtained from <https://bar.utoronto.ca/>

efp/cgi-bin/efpWeb.cgi?dataSource=Hormone&mode=Absolute&

primaryGene=At5g16780&secondaryGene=At3g27340&override=

&threshold=604.48&modeMask_low=None&modeMask_stddev=

None).
