# Supplementary material for: MDF Regulates a Network of Auxin‐Dependent and Auxin‐Independent Pathways of Adventitious Root Regeneration in Arabidopsis
Source: Plant Direct. 2025 Apr 23;9(4):e70050. doi: 10.1002/pld3.70050 (PMC12018534; doi:10.1002/pld3.70050)
Supplement: Supplementary file 4 — Table S3 PCR primers. [file PLD3-9-e70050-s001.docx]

**Table S3. PCR Primers**

| **Primer name** | **Sequence 5'-3'** |
| --- | --- |
| **Cloning:** |  |
| pDONR207 Forward | TCGCGTTAACGCTAGCATGGATCTC |
| pDONR207 Reverse | GTAACATCAGAGATTTTGAGACAC |
| *RAP2.7* Forward | GGGGACAAGTTTGTACAAAAAAGCAGGCTTAATGTTGGATCTTAACCTCAACGCT |
| *RAP2.7* Reverse | GGGGACCACTTTGTACAAGAAAGCTGGGTTTTAAGGGTGTGGATAAAAGTAACCACGT |
| *NAC1* Forward | GGGGACAAGTTTGTACAAAAAAGCAGGCTTAATGGAGACGGAAGAAGAGATGAAG |
| *NAC1* Reverse | GGGGACCACTTTGTACAAGAAAGCTGGGTTTCAGCAATTCCAAACAGTGCTTGGA |
| **RT-qPCR:** |  |
| *UBC* Forward | CTGCGACTCAGGGATCTTCTAA |
| *UBC* Reverse | TTGTGCCATTGAATTGAACCC |
| *UBQ10* Forward | GGCCTTGTATAATCCCTGATGAATAAG |
| *UBQ10*  Reverse | AAAGAGATAACAGGAACGGAAACATAGT |
| *ACT2*  Forward | CTTGCACCAAGCAGCATGAA |
| *ACT2*  Reverse | CCGATCCAGACACTGTACTTCCTT |
| *MDF* Forward | GGCCTGGAAAAATGAAGGA |
| *MDF* Reverse | GGCCACTGAGGACAAGGTAA |
| *PIN1* Forward | TCAGGGGAATAGTAACGACAACCAG |
| *PIN1* Reverse | ATCACACTTGTTGGTGGCATCACCT |
| *PIN3* Forward | ATTCCTCTCTACGTGGCCATGATCC |
| *PIN3* Reverse | AGAGAGGAGAGGGACGGCGAAG |
| *RAP2.7* Forward | TAATGGTAGAGAAGCAGTCACGAA |
| *RAP2.7* Reverse | TGGATAAAAGTAACCACGTGTTGC |
| *YUC1* Forward | TTCATGTGTTGCCAAGGGAGATAC |
| *YUC1* Reverse | ACCAATTTCGCCAGCGATCTTAAC |
| *NAC1* Forward | TGGGATGAGGAAGACATTGGTTTT |
| *NAC1* Reverse | TCAATCTTAGTGAGCTGACTGAGT |
| *WOX5* Forward | AAGCTTACGTGGCAACAATAACGG |
| *WOX5* Reverse | AAGATCTAATGGCGGTGGATGTTC |
| *IAA1*  forward | CGGTTAGATCTCACTGGAGGCCAT |
| *IAA1*  reverse | ACTTGCTCCTCCTCCTGCAAAAAC |
| *IAA2*  forward | AGGAAGAGTCTAGAGCAGGAGC |
| *IAA2*  reverse | ACTGGATGTTGGTTGGTGATG |
